# Conducting a Cluster RCT on Medication Safety in Nursing Units Overtaxed by the COVID-19 Pandemic

**DOI:** 10.1093/geroni/igab046.392

**Published:** 2021-12-17

**Authors:** Teryl Nuckols, Ed Seferian, Bernice Coleman, Carl Berdahl, Tara Cohen, Andrew Henreid

**Affiliations:** 1 Cedars-Sinai Medical Center, Los Angeles, California, United States; 2 Cedars-sinai Medical Center, Los Angeles, California, United States

## Abstract

Medication errors continue to harm many hospitalized patients. In other high-risk industries, voluntary incident reporting is widely used to improve safety. Reporting is widely used in hospitals, but not as effectively. This AHRQ-funded cluster RCT will assess the effects of the SAFE Loop, which includes five enhancements in incident reporting implemented on hospital nursing units. Analyses will compare changes in nurses’ attitudes toward reporting, event reporting rates, report quality, and medication event rates between intervention and control arms. The COVID-19 pandemic has created both obstacles and opportunities. The intervention requires study staff to engage nursing unit directors, attend daily nursing “huddles”, and train overtaxed front-line nurses in a geographic area greatly impacted by COVID-19 surges. This created uncertainty around the best time to start the trial. Conversely, we have collected unique data on the implications of COVID-19 for medication safety while testing our instruments during the trial preparation phase.

